# Brucellosis Surveillance — United States, 2010–2024

**DOI:** 10.15585/mmwr.ss7502a1

**Published:** 2026-06-04

**Authors:** Nekole S. Locke, Katherine M. DeBord, Rebekah V. Tiller, Robyn A. Stoddard, Elke Saile, Vit Kraushaar, Guillermo Adame, Connie Austin, Joel Ackelsberg, Alexandra Newman, Grishma Kharod, Marta Guerra, Rita M. Traxler, Meredith Parrado, William A. Bower, Caitlin M. Cossaboom, Dawn Blackburn, Alex R. Hoffmaster, Zachary Weiner, Maria E. Negron

**Affiliations:** ^1^Division of High-Consequence Pathogens and Pathology, CDC, Atlanta, Georgia; ^2^California Department of Public Health, Sacramento, California; ^3^Arizona Department of Health Services, Phoenix, Arizona; ^4^Illinois Department of Public Health, Springfield, Illinois; ^5^New York City Department of Health and Mental Hygiene, New York, New York; ^6^New York State Department of Health, Albany, New York

## Abstract

**Problem/Condition:**

Brucellosis is a zoonotic disease caused by bacteria of the *Brucella* genus, and humans can acquire the disease from infected animals or their by-products. This report on U.S. brucellosis cases is the first since the adoption of the 2010 Council of State and Territorial Epidemiologists’ (CSTE) brucellosis case definition.

**Period Covered:**

2010–2024.

**Description of System:**

Data from the National Notifiable Diseases Surveillance System (NNDSS), information from supplemental case report forms (CRFs), and data from analyses of clinical specimen testing by CDC during 2010–2024 were analyzed. The 2010 CSTE brucellosis case definition was used to determine case status using supplemental CRFs submitted for cases reported through NNDSS.

**Results:**

During 2010–2024, a total of 1,796 confirmed and probable cases of brucellosis were reported to CDC through NNDSS. A total of 878 of 1,796 (49%) cases also had a supplemental CRF submitted. Of these, 503 (57%) cases met the 2010 CSTE brucellosis confirmed or probable case definition on the basis of clinical and laboratory data submitted on the supplemental CRF; 383 cases (76%) were confirmed and 120 (24%) were probable. Among persons with brucellosis cases meeting the CSTE definition, 308 (61%) reported any travel, and of those, 245 (80%) reported only international travel during the 6 months before symptom onset. The most frequently reported exposures among persons with international travel were consumption of unpasteurized dairy products or undercooked meat (n = 195 [73%]). Among persons without international travel, the most frequently reported exposures were animal contact (n = 116 [62%]), particularly skinning and slaughtering wild animals (n = 47 [41%]) and hunting wild animals (n = 46 [40%]).

**Interpretation:**

The lack of adequate clinical or laboratory data led to the exclusion of nearly half of the supplemental CRFs from this analysis. However, the following recognized exposures to *Brucella* were identified in cases with adequate information: 1) consumption of unpasteurized dairy products or undercooked meat, particularly those produced outside the United States or meat from wild animals, and 2) animal contact, especially skinning and slaughtering of wild animals. Findings suggest ongoing public health messaging to reduce exposures from unpasteurized dairy products and undercooked meat, and to promote safe field dressing practices among hunters. For hunters and other persons who skin or slaughter wild animals, safe field dressing practices might help reduce infection risk.

**Public Health Action:**

Increasing brucellosis awareness among populations at risk for infection, health care providers, and public health officials will strengthen prevention and surveillance efforts and ultimately improve patient outcomes.

## Introduction

Brucellosis is a zoonotic disease caused by bacteria of the genus *Brucella*. Most human cases occur after contact with infected animals or their by-products (*1*). The primary *Brucella* species known to cause human disease are *Brucella melitensis*, *Brucella abortus*, *Brucella suis*, and *Brucella canis* (*2*). Brucellosis occurs worldwide, with an estimated annual incidence of 2.1 million human cases (*1*). Most cases occur in regions where the disease is highly endemic among animals, such as the Middle East, the Mediterranean basin, Central and South America, and parts of Africa and Asia (*3*). In the United States, rates of human infection declined during the 20th century after the completion of successful brucellosis eradication programs for domestic cattle and swine populations; however, *Brucella* species continue to circulate among wildlife, notably among feral swine in the southeastern United States and bison and elk in the Greater Yellowstone Area (*4*).

 Consuming unpasteurized dairy products or having direct contact with tissues or fluids from infected animals have been identified as common forms of exposure to *Brucella *species. *Brucella* bacteria can be easily aerosolized and inhaled. Persons involved in certain occupations or activities (e.g., slaughterhouse work, laboratory work, veterinary practice, and hunting) are at greater risk for exposure (*5*). Human-to-human transmission is possible but occurs rarely: in utero or through sexual contact, breastfeeding, organ transplants, or blood transfusions (*6*).

The clinical signs and symptoms of brucellosis are nonspecific, with fever being the most common. More severe complications include neurologic symptoms, endocarditis, and musculoskeletal manifestations. The case-fatality rate is <5%, with most deaths associated with endocarditis (*7*).

The diagnostic gold standard for brucellosis is culturing *Brucella* bacteria from blood, cerebrospinal fluid or joint fluid, abscess, or tissue specimens. However, serologic tests often are used because they are readily available and provide results in less time. These tests detect antibodies only against smooth *Brucella* species* (e.g., *B. melitensis*, *B. suis*, and *B. abortus)* (*8*). Infections caused by rough *Brucella* species (e.g., *B. canis* and *B. abortus* vaccine strain RB51 [*B. abortus* RB51]) can only be definitively diagnosed by culture results. Polymerase chain reaction (PCR) testing of isolates often is used to identify species but is not widely available or routinely used in the United States to analyze clinical specimens because of a high false-negative rate resulting from low bacillary levels. In certain patients with brucellosis, *Brucella* bacteria might continue to be detectable by PCR testing after the patients complete treatment because of the persistence of the pathogen’s DNA in their blood (*9*,*10*).

The antimicrobial treatment most often used for uncomplicated brucellosis is a combination of doxycycline and rifampin. However, other antimicrobial regimens might be considered due to the presence of certain complicated conditions (e.g., spondylitis, neurobrucellosis, and endocarditis), drug allergies or contraindications, and other factors (e.g., patient age, *B. abortu*s RB51 infection, or pregnancy) (*11*–*13*). Patients might relapse because of lack of treatment adherence or inadequate management (e.g., monotherapy or insufficient duration of treatment).

In the United States, the Council of State and Territorial Epidemiologists (CSTE) establishes case definitions for nationally notifiable conditions, including brucellosis (*14*). CSTE updated the brucellosis case definition in 2010, and since that update no reports have been published that summarize U.S. cases. This report summarizes U.S. brucellosis cases reported to CDC during 2010–2024, describing patient demographic characteristics, clinical presentation, exposures, and laboratory testing.

## Methods

### Data Sources and Collection

Brucellosis is a nationally notifiable condition and is reportable in all 57 U.S. reporting jurisdictions; however, reporting requirements (i.e., who reports, how reports are received, and timing) vary by jurisdiction. Jurisdictions voluntarily submit non–disease-specific case data (e.g., case status, patient demographic information, and reporting jurisdiction) to CDC through the National Notifiable Diseases Surveillance System (NNDSS) for all nationally notifiable conditions. For brucellosis cases, jurisdictions can also submit supplemental data to CDC using the brucellosis case report form (CRF), which includes clinical information, laboratory data, outcomes, and exposures. CRFs are submitted to CDC by secure email, fax, mail, or the One CDC Data Platform (1CDP). The 2020 national and jurisdictional population estimates were obtained from the U.S. Census Bureau (*15*).

### Data Analysis

The 2010 CSTE brucellosis case definition was used for this analysis (*14*). NNDSS data analyzed included case count and case classification reported by jurisdiction, sex, age, race, and ethnicity. Jurisdictions determine case definitions used to report to NNDSS based on their local mandates. Total case counts from NNDSS during 2010–2024 and the national and jurisdiction-specific population estimates for 2020 were used to determine the 15-year cumulative brucellosis incidence over the study period. Incidence maps were created in R software (version 4.2.3; R Foundation).

Supplemental CRF data analyzed included patient signs and symptoms, antibiotic treatment, travel in the 6 months before symptom onset, animal contact, animal product consumption, laboratory test types, and outcomes. The 2010 CSTE brucellosis case definition was used to determine case classification based on the clinical and laboratory criteria reported on CRFs. All cases with CRF data that did not meet CSTE case classification criteria, including those with missing clinical or laboratory data, were excluded from these analyses. Comparisons among exposure groups between persons with reported international travel and no reported international travel were conducted using chi-square tests, with statistical significance defined as two-sided p-values <0.05; cases with missing or unknown travel information were excluded. All descriptive analyses were performed using 1CDP and statistical analyses were conducted using SAS software (version 9.4; SAS Institute). This activity was reviewed by CDC, deemed not research, and conducted consistent with applicable federal law and CDC policy.^†^

### *Brucella* Species Testing Performed at CDC

Certain U.S. laboratories, including clinical and state public health laboratories, submit isolates to CDC for species identification and additional testing for surveillance purposes. *Brucella* isolates submitted to CDC during the study period were identified at the species level using the Laboratory Response Network testing algorithm (*16*), which includes a combination of real-time PCR and biochemical tests. In addition, *Brucella* microagglutination tests (BMATs) were performed on serology specimens sent to CDC during the study period. 

## Results

### CDC National Notifiable Diseases Surveillance System

**FIGURE 1:**
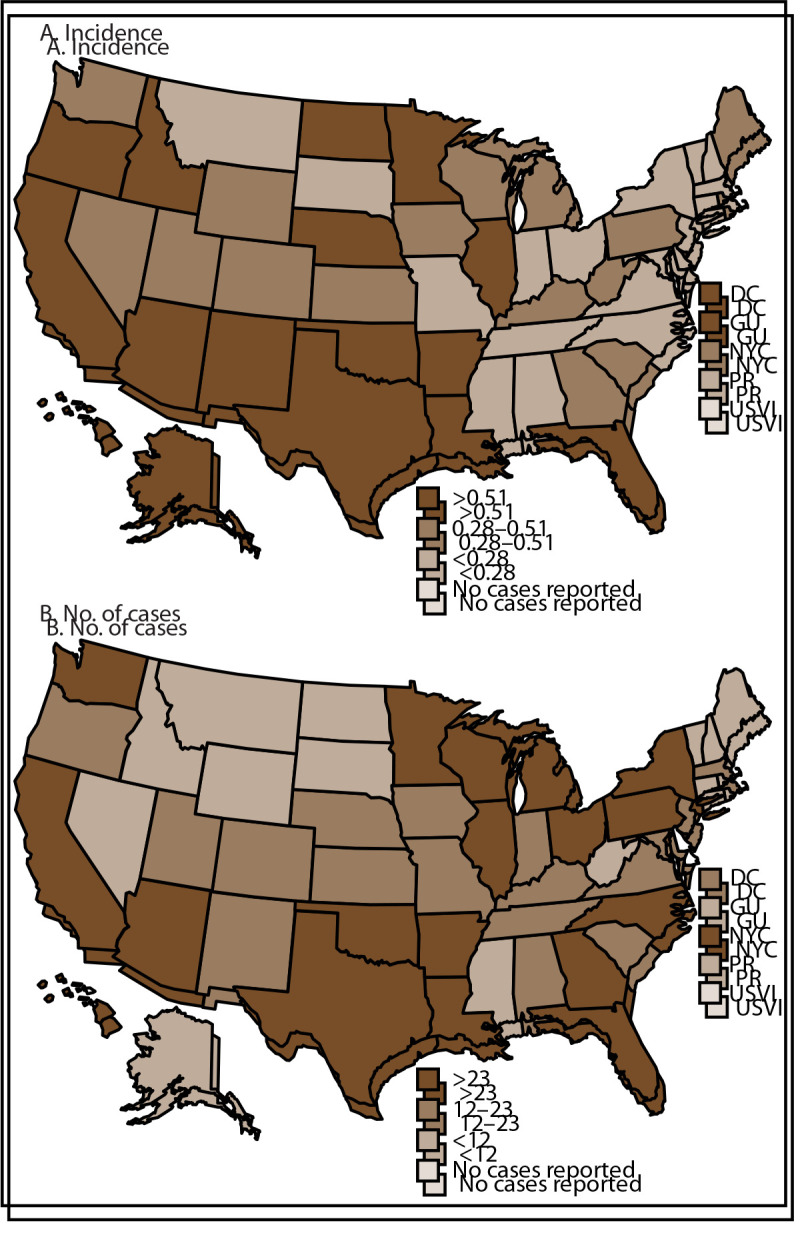
Fifteen-year cumulative incidence*^,†^ (A) and number of brucellosis cases^§^ (B), by jurisdiction — National Notifiable Diseases Surveillance System, United States, 2010–2024 **Abbreviations:** DC = District of Columbia; GU = Guam; NYC = New York City; PR = Puerto Rico; USVI = U.S. Virgin Islands. * Per 100,000 population. ^†^ Cumulative incidence was calculated using total reported brucellosis cases during 2010–2024 divided by the U.S. Census Bureau (https://data.census.gov) 2020 population estimate. ^§^ Case counts for reporting year 2024 are provisional and subject to change.

During 2010–2024, CDC was notified through NNDSS of 1,796 brucellosis cases from 54 jurisdictions. California (n = 371 [21%]; 15-year cumulative incidence = 0.94 per 100,000 population), Texas (n = 327 [18%]; 15-year cumulative incidence = 1.13), and Florida (n = 128 [7%]; 15-year cumulative incidence = 0.59) reported the most cases (Figure 1). Jurisdictions reported 71–165 cases annually during 2010–2024 (Supplementary Figure 1). Seventy-seven percent (n = 1,377) of all cases reported through NNDSS were classified as confirmed, and 23% (n = 419) were classified as probable (Table 1). More than half of all cases reported were among males (n = 1,069 [60%]), and the median age of persons with cases was 49 years (IQR range = 32–64 years). Overall, 57% (n = 1,026) of reported cases were among White persons, and 49% (n = 875) were among Hispanic or Latino persons.

**TABLE 1:** Demographic characteristics of patients with brucellosis cases, by jurisdiction-reported case classification — National Notifiable Diseases Surveillance System, United States, 2010–2024*

Characteristic	Confirmed cases (n = 1,377)	Probable cases (n = 419)	Total cases (N = 1,796)
	No. (%)	No. (%)	No. (%)
**Sex**			
Female	516 (37.5)	201 (48.0)	**717 (39.9)**
Male	853 (61.9)	216 (51.6)	**1,069 (59.5)**
Unknown	8 (0.6)	2 (0.5)	**10 (0.6)**
**Age group, yrs**			
Median (IQR)	50 (33–64)	42 (30–59)	**49 (32–64)**
<1	4 (0.3)	0 (—)	**4 (0.2)**
1–9	60 (4.4)	10 (2.4)	**70 (3.9)**
10–19	74 (5.4)	32 (7.6)	**106 (5.9)**
20–29	143 (10.4)	62 (14.8)	**205 (11.4)**
30–39	196 (14.2)	83 (19.8)	**279 (15.5)**
40–49	190 (13.8)	71 (16.9)	**261 (14.5)**
50–59	221 (16.0)	58 (13.8)	**279 (15.5)**
60–69	268 (19.5)	58 (13.8)	**326 (18.2)**
≥70	220 (16.0)	44 (10.5)	**264 (14.7)**
Unknown	1 (0.1)	1 (0.2)	**2 (0.1)**
**Race**			
American Indian or Alaska Native	21 (1.5)	4 (1.0)	**25 (1.4)**
Asian, Native Hawaiian, or Other Pacific Islander	67 (4.9)	12 (2.9)	**79 (4.4)**
Black or African American	87 (6.3)	12 (2.9)	**99 (5.5)**
White	764 (55.5)	262 (62.5)	**1,026 (57.1)**
Other	204 (14.8)	33 (7.9)	**237 (13.2)**
Unknown	243 (17.6)	96 (22.9)	**339 (18.9)**
**Ethnicity**			
Hispanic or Latino	750 (54.5)	125 (29.8)	**875 (48.7)**
Not Hispanic or Latino	448 (32.5)	180 (43.0)	**628 (35.0)**
Unknown	179 (13.0)	114 (27.2)	**293 (16.3)**

* Case counts for reporting year 2024 are provisional and subject to change.

### Supplemental Case Report Form Data

**FIGURE 2:**
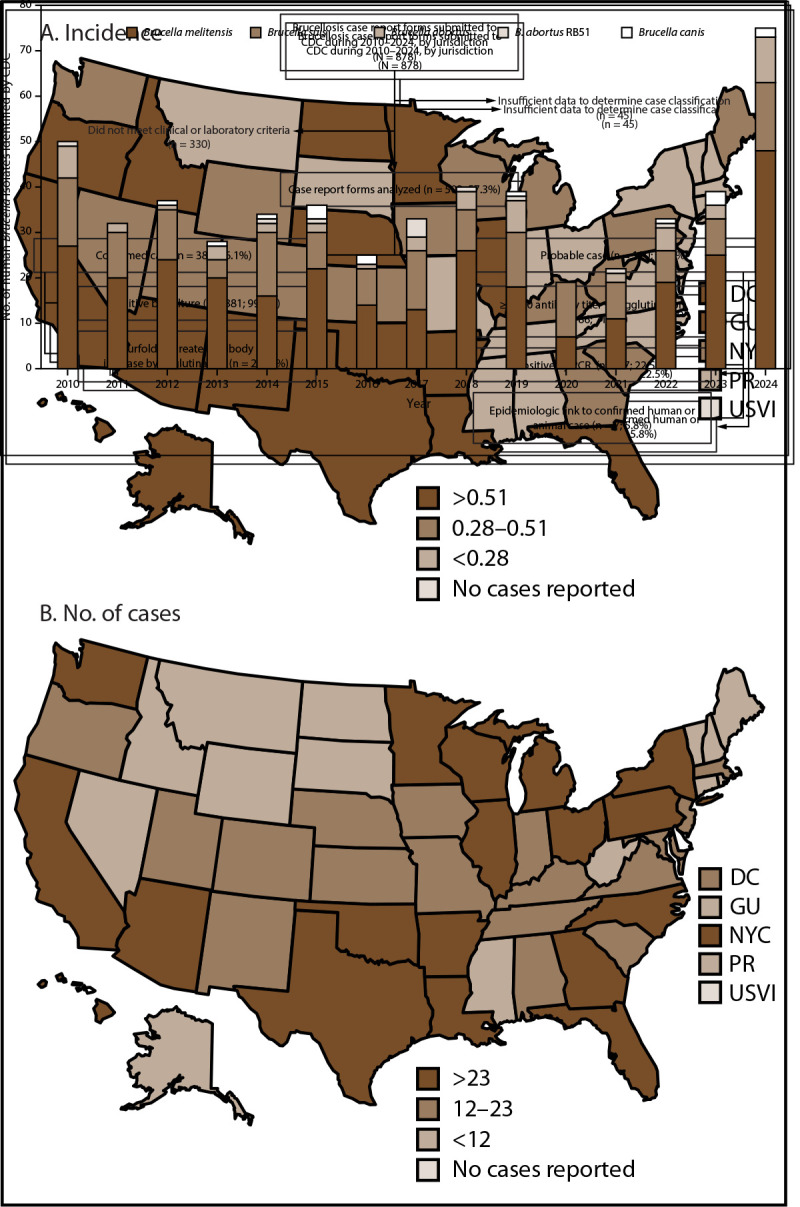
Brucellosis case report form data, by case classification of the Council of State and Territorial Epidemiologists — United States, 2010–2024* **Abbreviation:** PCR = polymerase chain reaction. * Clinical and laboratory criteria as described in the Council of State and Territorial Epidemiologists’ 2010 brucellosis case definition. https://ndc.services.cdc.gov/case-definitions/brucellosis-2010/

Jurisdictions submitted supplemental CRFs for 49% (n = 878) of cases reported through NNDSS during 2010–2024. Of the 878 supplemental CRFs received, 38% (n = 330) did not meet clinical or laboratory criteria to be classified as confirmed or probable cases (Figure 2). Culture confirmation without meeting clinical criteria requirements was the most common scenario for not meeting the 2010 CSTE brucellosis case definition (n = 219 [66%]). Lack of reported fever (n = 277 [84%]) was the most common reason that cases did not meet the clinical criteria. Twenty-one cases (6%) did not meet the CSTE case definition because of a lack of laboratory evidence (e.g., enzyme-linked immunosorbent assay [ELISA] testing only). Forty-five (5%) CRFs were excluded because of insufficient data to determine case classification.

### Confirmed Brucellosis Cases

Of the 503 cases with supplemental CRFs that met the 2010 CSTE brucellosis case definition, 383 (76%) were classified as confirmed cases. All but two of these patients (n = 381 [99%]) received a diagnosis that was confirmed by culture results; the remaining two patients (0.5%) received confirmation by agglutination testing (Figure 2). Sixty percent (n = 229) of culture-confirmed cases reported also having a PCR test result for an isolate or a clinical specimen; 97% (n = 221) of these cases had positive PCR test results. In addition, 21% (n = 82) of culture-confirmed cases were reported in patients who had agglutination tests performed; 83% (n = 68) of these were positive. Thirty-five (9%) patients with culture-confirmed cases reported having an ELISA test, and the majority of results (n = 31 [89%]) were positive for either immunoglobulin M (IgM) or immunoglobulin G (IgG). Of the two patients with confirmed cases for whom a fourfold antibody titer increase was detected, one also received ELISA testing and the results were positive for IgM and IgG (Supplementary Figure 2).

In addition to fever, which was required for a patient’s illness to be classified as brucellosis, fatigue (n = 264 [69%]), night sweats (n = 227 [59%]), headache (n = 185 [48%]), and anorexia (n = 182 [48%]) were the most commonly reported symptoms among patients with confirmed cases (Table 2). Arthritis, spondylitis, or arthralgia was reported among 49% (n = 189) of patients. Seventy-five percent (n = 288) of patients with confirmed cases were hospitalized, and <1% (n = three) died. Of the 383 confirmed cases, 80% (n = 307) were among patients reported to have been administered or prescribed antibiotic treatment, 19% (n = 73) were missing treatment information, and <1% (n = three) were reported to have received no antimicrobial treatment. Of the 307 patients who were prescribed or administered antimicrobials, 175 (57%) received two antimicrobials, and 28 (9%) were prescribed or administered a single antimicrobial. Doxycycline (n = 254 [83%]) and rifampin (n = 191 [62%]) were the most reported antimicrobials; 45% (n = 174) of patients with confirmed cases who received antimicrobials (n = 307) received both. Eight patients with confirmed cases reported receiving streptomycin; all eight were also prescribed doxycycline, rifampin, or both.

**TABLE 2:** Clinical characteristics and treatment information for patients with brucellosis cases* with completed case report forms — United States, 2010–2024

Characteristic	CSTE case definition			Total no. of submitted CRFs with sufficient data to determine case classification (n = 833)
	Confirmed (n = 383)	Probable (n = 120)	Did not meet criteria (n = 330)	
	No. (%)	No. (%)	No. (%)	No. (%)
**Hospitalized**				
Yes	288 (75.2)	70 (58.3)	125 (37.9)	**483 (58.0)**
No	62 (16.2)	38 (31.7)	61 (18.5)	**161 (19.3)**
Unknown	3 (0.8)	3 (2.5)	5 (1.5)	**11 (1.3)**
Missing	30 (7.8)	9 (7.5)	139 (42.1)	**178 (21.4)**
**Died**				
Yes	3 (0.8)	3 (2.5)	6 (1.8)	**12 (1.4)**
No	275 (71.8)	85 (70.8)	136 (41.2)	**496 (59.5)**
Unknown	6 (1.6)	1 (0.8)	3 (0.9)	**10 (1.2)**
Missing	99 (25.8)	31 (25.8)	185 (56.1)	**315 (37.8)**
**Sign or symptom**				
Fever or chills	383 (100)	120 (100)	98 (29.7)	**601 (72.1)**
Fatigue	264 (68.9)	81 (67.5)	71 (21.5)	**416 (49.9)**
Night sweats	227 (59.3)	62 (51.7)	53 (16.1)	**342 (41.1)**
Headache	185 (48.3)	69 (57.5)	51 (15.5)	**305 (36.6)**
Myalgia	176 (46.0)	66 (55.0)	43 (13.0)	**285 (34.2)**
Anorexia	182 (47.5)	54 (45.0)	33 (10.0)	**269 (32.3)**
Arthralgia	140 (36.6)	53 (44.2)	55 (16.7)	**248 (29.8)**
Weight loss	137 (35.8)	40 (33.3)	37 (11.2)	**214 (25.7)**
Gastrointestinal symptom^†^	74 (19.3)	17 (14.2)	28 (8.5)	**119 (14.3)**
Focal organ involvement^§^	74 (19.3)	21 (17.5)	12 (3.6)	**107 (12.8)**
Arthritis or spondylitis	49 (12.8)	15 (12.5)	24 (7.3)	**88 (10.6)**
Respiratory symptom^¶^	37 (9.7)	19 (15.8)	12 (3.6)	**68 (8.2)**
Meningitis	5 (1.3)	1 (0.8)	1 (0.3)	**7 (0.8)**
Other	142 (37.1)	43 (35.8)	110 (33.3)	**295 (35.4)**
No data reported	0 (—)	0 (—)	102 (30.9)	**102 (12.2)**
**Antibiotic use**				
Yes	307 (80.2)	97 (80.8)	140 (42.4)	**544 (65.3)**
No	3 (0.8)	3 (2.5)	9 (2.7)	**15 (1.8)**
Missing	73 (19.1)	20 (16.7)	181 (54.8)	**274 (32.9)**
**Antibiotic**				
Doxycycline	254 (82.7)	80 (82.5)	105 (75.0)	**439 (80.7)**
Rifampin	191 (62.2)	52 (53.6)	73 (52.1)	**316 (58.1)**
Aminoglycoside**	102 (33.2)	22 (22.7)	34 (24.3)	**158 (29.0)**
Ceftriaxone	31 (10.1)	6 (6.2)	14 (10.0)	**51 (9.4)**
Ciprofloxacin	25 (8.1)	6 (6.2)	5 (3.6)	**36 (6.6)**
Vancomycin	19 (6.2)	6 (6.2)	11 (7.9)	**36 (6.6)**
Trimethoprim or sulfamethoxazole	19 (6.2)	5 (5.2)	12 (8.6)	**36 (6.6)**
Other	40 (13.0)	22 (22.7)	19 (13.6)	**83 (15.3)**
**Number of antibiotics^††^**				
1	28 (9.1)	18 (18.6)	28 (20.0)	**74 (13.6)**
2	175 (57.0)	51 (52.6)	64 (45.7)	**290 (53.3)**
3	67 (21.8)	17 (17.5)	25 (17.9)	**109 (20.0)**
Four or more	25 (8.1)	8 (8.2)	10 (7.1)	**43 (7.9)**

**Abbreviations:** CRF = case report form; CSTE = Council of State and Territorial Epidemiologists.

* According to the 2010 CSTE brucellosis case definition.

^†^ Includes vomiting, nausea, diarrhea, abdominal pain, abdominal bloating, and constipation.

^§^ Includes endocarditis, orchitis, epididymitis, hepatomegaly, and splenomegaly.

^¶^ Includes cough, upper respiratory infection, respiratory symptoms, sinus pain, nasal congestion, sore throat, shortness of breath, and pneumonia.

** Reported aminoglycosides include gentamicin, streptomycin, amikacin, and tobramycin.

^††^ Information on the timing of antibiotics was not available, so whether antibiotics were taken in combination or in sequence is unknown.

### Probable Brucellosis Cases

Twenty-four percent (n = 120) of the 503 cases with a supplemental CRF that met the 2010 CSTE case definition were classified as probable (Figure 2). Of these, 94% (n = 113) were among patients with presumptive laboratory testing for brucellosis (i.e., a single positive agglutination test result or positive PCR test result for a clinical specimen), and 6% (n = 7) had an epidemiologic linkage to a confirmed human or animal case of brucellosis. Of the 113 patients with positive presumptive laboratory testing data, a single positive agglutination test result was reported for 71% (n = 80), a positive PCR test result for 24% (n = 27), and both positive agglutination test results and positive PCR test results were reported for 5% (n = 6) (Supplementary Figure 3). Thirty-five percent (n = 28) of the 80 patients with probable cases who had a single positive agglutination test result were also reported to have had an ELISA test; all but one (n = 27 [96%]) received positive ELISA test results for either IgG or IgM. Three (11%) of the 27 patients who received PCR testing also received a positive ELISA test result: two for IgG and one for IgM. No cases were identified among patients with both positive agglutination and PCR test results who also had ELISA testing performed. Five of the seven patients (71%) with an epidemiologic linkage were also reported to have had ELISA testing; all ELISA test results were IgM-positive, and one (20%) also was IgG-positive.

Other than fever, fatigue (n = 81 [68%]), headache (n = 69 [58%]), and myalgia (n = 66 [55%]) were the most frequently reported symptoms among persons with probable cases (Table 2). Arthralgia (44%) and arthritis or spondylitis (13%) were commonly reported symptoms. Fifty-eight percent (n = 70) of persons with probable cases were hospitalized, and 3% (n = three) died. Of the persons with probable cases with reported data on antimicrobial treatment, all but three (n = 97 of 100 [97%]) were administered or prescribed treatment; 18 (19%) received a single antimicrobial. Doxycycline (n = 80 [83%]) and rifampin (n = 52 [54%]) were the two most frequently prescribed antimicrobials; 41% (n = 49) of patients with probable cases who received antimicrobials received both. One patient with a probable case received streptomycin.

### Exposures

In the 6 months before symptom onset, approximately one half of confirmed and probable cases with CRF data (n = 308 [61%]) were among persons who reported travel (Table 3). Most indicated traveling outside the United States (n = 245 [80%]) rather than domestically (n = 37 [12%]), and 23 persons (8%) reported traveling both internationally and domestically. Mexico (n = 155 [58%]) was the most frequently reported international travel location.

**TABLE 3:** Reported travel 6 months before illness onset among patients with brucellosis cases* with completed case report forms — United States, 2010–2024

Characteristic	Confirmed cases (n = 383)	Probable cases (n = 120)	Total cases (N = 503)
	No. (%)	No. (%)	No. (%)
**Any reported travel**			
Yes	238 (62.1)	70 (58.3)	**308 (61.2)**
No	113 (29.5)	36 (30.0)	**149 (29.6)**
Unknown	24 (6.3)	10 (8.3)	**34 (6.8)**
Missing	8 (2.1)	4 (3.3)	**12 (2.4)**
**Travel location^†^**			
Domestic only	28 (11.8)	9 (12.9)	**37 (12.0)**
International only	191 (80.3)	54 (77.1)	**245 (79.5)**
Domestic and international	18 (7.6)	5 (7.1)	**23 (7.5)**
Missing	1 (0.4)	2 (2.9)	**3 (1.0)**
**Region of international travel**			
Africa	24 (11.5)	6 (10.2)	**30 (11.2)**
Europe	11 (5.3)	6 (10.2)	**17 (6.3)**
Latin America (excluding Mexico)	14 (6.7)	8 (13.6)	**22 (8.2)**
Mexico	126 (60.3)	29 (49.2)	**155 (57.8)**
Middle East	30 (14.4)	10 (16.9)	**40 (14.9)**
Southeast Asia	6 (2.9)	3 (5.1)	**9 (3.4)**
Western Asia and Pacific	2 (1.0)	1 (1.7)	**3 (1.1)**

* According to the 2010 Council of State and Territorial Epidemiologists’ brucellosis case definition.

^†^ Travel region categories are not mutually exclusive.

Of all confirmed or probable cases with CRF data, 229 (46%) were among patients reporting animal contact, and the majority (n = 285 [57%]) reported consuming unpasteurized dairy products or undercooked meat (Table 4). Overall, among cases without international travel, more patients were reported to have had contact with animals (n = 116 [62%]) than those who reported international travel (n = 99 [37%]; p = 0.001). Forty-seven percent (n = 54) of patients with animal contact but no international travel reported contact with feral swine, and 38% (n = 44) reported contact with dogs. Skinning or slaughtering (n = 47 [41%]) and hunting (n = 46 [40%]) wild animals (e.g., feral swine and deer) were the two most frequently reported types of animal contact among patients with no international travel. Drinking unpasteurized milk was more common among patients with international travel (n = 100 [51%]; p<0.001) and eating raw or undercooked meat was more common among patients without international travel (n = 17 [22%]; p = 0.002). Regardless of reported travel, among patients who reported consuming an unpasteurized dairy product or undercooked meat, 75% (n = 214) reported the product was from outside the United States. Of the 38 patients with brucellosis cases who reported consuming a product from the United States, three also reported consuming a product of international origin. Of the remaining 35 cases, 14 (40%) were among patients who reported consuming unpasteurized milk or soft cheese from a goat; 10 (29%) reported eating undercooked wild animal meat (e.g., feral swine, deer, elk, or reindeer); seven (20%) reported consuming unpasteurized milk, soft cheese, or unpasteurized yogurt from cattle; and four (11%) reported eating soft cheese from an unspecified animal.

**TABLE 4:** Reported animal contact, animal product consumption, and international travel 6 months before illness onset among persons with brucellosis cases* with completed case report forms — United States, 2010–2024

Characteristic	International travel (n = 268)	No international travel (n = 186)	Unknown or missing travel (n = 49)	Total cases (N = 503)
	No. (%)	No. (%)	No (%)	No. (%)
**Animal contact during previous 6 months**				
Animal contact				
Yes	99 (36.9)	116 (62.4)	14 (28.6)	**229 (45.5)**
No	105 (39.2)	62 (33.3)	5 (10.2)	**172 (34.2)**
Unknown	36 (13.4)	3 (1.6)	23 (46.9)	**62 (12.3)**
Missing	28 (10.4)	5 (2.7)	7 (14.2)	**40 (8.0)**
Animal				
Cow	36 (36.4)	18 (15.5)	2 (14.3)	**56 (24.5)**
Deer or elk	1 (1.0)	15 (12.9)	1 (7.1)	**17 (7.4)**
Dog	30 (30.3)	44 (37.9)	2 (14.3)	**76 (33.2)**
Goat or sheep	36 (36.4)	17 (14.7)	2 (14.3)	**55 (24.0)**
Feral swine	17 (17.2)	54 (46.6)	9 (64.3)	**80 (34.9)**
Other^†^	33 (33.3)	31 (26.7)	2 (14.3)	**66 (28.8)**
Missing	13 (13.1)	6 (5.2)	1 (7.1)	**20 (8.7)**
Contact type				
Birthing	12 (12.1)	11 (9.5)	0 (—)	**23 (10.0)**
Hunting	2 (2.0)	46 (39.7)	8 (57.1)	**56 (24.5)**
Skinning or slaughtering	13 (13.1)	47 (40.5)	5 (35.7)	**65 (28.4)**
General	39 (39.4)	36 (31.0)	4 (28.6)	**79 (34.5)**
Other	20 (20.2)	23 (19.8)	2 (14.3)	**45 (19.7)**
Missing	24 (24.2)	11 (9.5)	1 (7.1)	**36 (15.7)**
**Consumption of unpasteurized dairy, raw or soft cheese, or raw or undercooked meat during previous 6 months**				
Consumption of unpasteurized dairy, raw or soft cheese, or raw or undercooked meat				
Yes	195 (72.8)	76 (40.9)	14 (28.6)	**285 (56.7)**
No	35 (13.1)	90 (48.4)	5 (10.2)	**130 (25.8)**
Unknown	32 (11.9)	14 (7.5)	24 (49.0)	**70 (13.9)**
Missing	6 (2.2)	6 (3.2)	6 (12.2)	**18 (3.6)**
Product				
Unpasteurized milk	100 (51.3)	9 (11.8)	5 (35.7)	**114 (40.0)**
Raw or soft cheese	114 (58.5)	50 (65.8)	11 (78.6)	**175 (61.4)**
Raw or undercooked meat	17 (8.7)	17 (22.4)	1 (7.1)	**35 (12.3)**
Other	4 (2.1)	0 (—)	0 (—)	**4 (1.4)**
Missing	12 (6.2)	5 (6.6)	0 (—)	**17 (6.0)**
Product origin				
United States	10 (5.1)	26 (34.2)	2 (14.3)	**38 (13.3)**
International	167 (85.6)	39 (51.3)	8 (57.1)	**214 (75.1)**
Missing	20 (10.3)	12 (15.8)	4 (28.6)	**36 (12.6)**
Animal source				
Camel	22 (11.3)	0 (—)	0 (—)	**22 (7.7)**
Cow	69 (35.4)	26 (25.4)	2 (14.3)	**97 (34.4)**
Goat or sheep	66 (33.8)	23 (30.3)	4 (28.6)	**93 (32.6)**
Feral swine	7 (3.6)	8 (10.5)	0 (—)	**15 (5.3)**
Other^§^	6 (3.1)	7 (9.2)	1 (7.1)	**14 (4.9)**
Unknown	50 (25.6)	16 (21.1)	7 (50.0)	**73 (25.6)**
Missing	24 (12.3)	5 (6.6)	0 (—)	**29 (10.2)**

* According to the 2010 Council of State and Territorial Epidemiologists’ brucellosis case definition.

^†^ Other animals included camel, bison, bear, caribou, reindeer, horse, donkey, llama, cat, rabbit, chicken, duck, turkey, ferret, guinea pig, porpoise, kangaroo, lemur, wallaby, snake, raccoon, skunk, and squirrel.

^§^ Other animal sources included deer, elk, bison, reindeer, turkey, chicken, rodent, and turtle.

### *Brucella* Species Testing Performed at CDC

**FIGURE 3:**
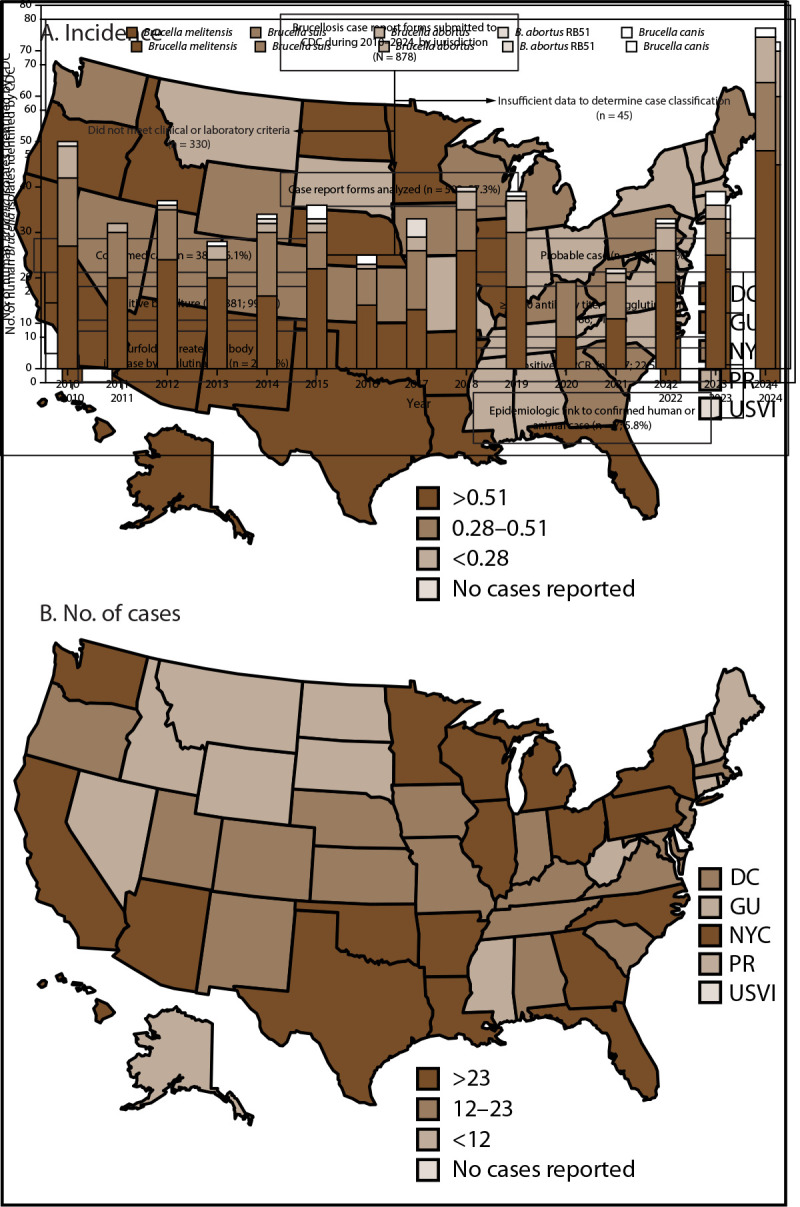
Number of human *Brucella* isolates identified by CDC, by year — United States, 2010–2024

During 2010–2024, CDC performed confirmatory testing on 542 human *Brucella* species isolates from 44 jurisdictions. Overall, *B. melitensis* (n = 310 [57%]) was the most frequently identified species, followed by *B. suis* (n = 154; [28%]) (Figure 3). Eleven isolates (2%) were identified as *B. abortus* RB51 and 15 (3%) were identified as *B. canis*. Because isolate submission to CDC is not population-based, these data should not be interpreted as the species distribution for all U.S. brucellosis cases.

In addition, CDC performed BMATs on 1,735 specimens from 1,540 patients during 2010–2024. Of the tested specimens, 140 (8%) were seropositive (*Brucella* species antibody titers ≥1:160). Among 172 patients (11%) who had more than one specimen tested, three patients (2%) had a fourfold or greater increase in antibody titers between acute and convalescent specimens.

## Discussion

A review published in 2008 that summarized NNDSS human brucellosis case reports during 1951–2001 highlighted a significant decline in the 1960s from more than 6,000 cases per year to 100–150 cases per year, followed by decades of stability in case prevalence (*4*). This decline was attributed to the implementation of effective control measures such as the Cooperative State-Federal Brucellosis Eradication Program (*17*) and the widespread pasteurization of dairy products (*4*). The findings in this report align with that 2008 review, demonstrating that annual human brucellosis cases reported through NNDSS remained relatively stable but persistent at 71–165 cases annually during 2010–2024. To explain this continued level of brucellosis transmission, an analysis of supplemental CRF data identified two exposures which could put persons in the United States at risk for brucellosis: 1) consumption of unpasteurized dairy products sourced from outside the United States and 2) direct contact with wild animals in the United States, particularly among hunters and persons involved in the slaughtering or skinning of animals.

Among analyzed cases with supplemental CRFs, reported consumption of unpasteurized dairy products or undercooked meat was common, particularly among those with international travel. Unpasteurized dairy products imported from outside the United States have been linked to multiple U.S. brucellosis outbreaks (*18*). Although rare, U.S.-produced, unpasteurized products have also been associated with brucellosis cases, often as a result of infection with the cattle vaccine strain *B. abortus* RB51 (*19*–*21*). This vaccine is a modified live vaccine that is used in the United States. The vaccine is typically cleared from the animal’s bloodstream within 3 days of vaccination, although it might occasionally be persistently shed by the animal in milk after vaccination (*22*). Thus, continuing to highlight the risks for consuming unpasteurized dairy products, both from within and outside the United States, is essential for prevention of brucellosis infection.

A second pattern in the analyzed data was reported animal contact among persons without international travel, particularly during hunting-related activities and skinning or slaughtering of wild animals; feral swine was the most frequently reported animal exposure in this subgroup. A small number of interactions were reported with deer, elk, and caribou. Although brucellosis was eradicated from the U.S. domestic swine population in 2011, the disease remains prevalent among feral swine (*23*). As the geographic range of feral swine expands in the United States, the exposure risk for hunters increases (*24*). To reduce this risk, hunters should practice safe field dressing techniques, such as wearing latex or rubber disposable gloves, using eye protection, and avoiding contact with animal fluids or organs (*25*).

One third of the persons with brucellosis cases in this analysis with reported animal contact indicated contact with dogs; however, this observation is difficult to interpret for multiple reasons. First, because approximately 45% of U.S. households own a dog (*26*), determining the significance of dog contact is difficult. Second, because of limitations in the data, whether these dogs were infected with *Brucella* bacteria is unknown. Third, human cases of *B. canis* in the United States are believed to be underreported because of 1) a lack of awareness about the disease and 2) challenges with the availability of reliable diagnostic tests (*27*); no serologic tests are available in the United States for detecting human antibodies against *B. canis*. Finally, humans appear to be relatively resistant to infection with *B. canis* and might experience milder disease than they do with infection by other *Brucella* species (*28*).

Brucellosis symptoms often are nonspecific, leading to underdiagnosis, underreporting, and delays in treatment. Among the confirmed and probable cases analyzed, approximately 40% were among persons who reported arthralgia, and 13% reported arthritis or spondylitis, which are lower than previously reported rates (65% and 26%, respectively) (*29*). The surveillance system recorded six deaths (1%), consistent with the 1%–2% mortality range previously reported (*8*,*9*); however, because death status was missing for a substantial proportion of CRFs, this finding should not be interpreted as a definitive case-fatality estimate.

Most patients received multiple antimicrobials, with doxycycline and rifampin being the most frequently reported combination. This combination often is used for treating uncomplicated brucellosis because of its ease of administration (i.e., oral) (*30*). In contrast, only six persons received doxycycline and streptomycin, likely because of the logistic challenges of administering streptomycin intramuscularly (*30*). In addition, 14% of persons were treated with a single antimicrobial. Monotherapy for brucellosis is associated with high relapse rates (approximately 50%) and is generally considered inadequate (*30*).

The 2010 CSTE brucellosis case definition required fever as part of the clinical criteria for a case, which not only complicates the interpretation of clinical symptom data but also reduces the sensitivity of brucellosis surveillance; approximately 37% of persons with culture-confirmed cases of brucellosis were excluded from this analysis because of the absence of reported fever. Certain excluded persons likely had unrecorded fevers, and others might not have developed a fever despite positive *Brucella* bacterial cultures. Because culture is considered the gold standard for brucellosis diagnosis, in 2024, CSTE removed fever as a clinical criterion among persons with culture-positive results in its updated case definition position statement, which went into effect in January 2025 (*31*).

All but two confirmed cases were among patients with *Brucella*-positive cultures, which aligns with the gold standard for brucellosis diagnosis (*8*,*32*). Approximately 25% of patients with culture results yielding *Brucella *bacteria also had a serology test performed, and 5% of persons with probable cases diagnosed after a single agglutination test result also had a positive PCR test result, indicating that clinicians are requesting multiple testing modalities for patient diagnosis. CDC conducts serologic testing using BMATs, and several commercial laboratories in the United States offer *Brucella* species ELISA and reflex agglutination testing services; however, commercial laboratories use different test kits with varying performance characteristics, making data comparison challenging.

Overall, agglutination testing was reported twice as often as ELISA testing among the culture-confirmed cases, and 67% of patients with probable cases were diagnosed on the basis of a single positive agglutination test result. The 2010 CSTE brucellosis case definition did not include ELISA testing among the laboratory criteria, likely influencing the higher frequency and reporting of agglutination testing. *Brucella* species agglutination testing is recommended most often for persons with acute, noncomplicated cases (*8*,*33*).

Of the 35 patients with positive cultures and ELISA testing, 31 (89%) received a positive ELISA result (24 positive for either IgM or IgG and seven positive for both IgM and IgG), suggesting that ELISA antibody detection was common among this small number of culture-confirmed cases (*34*). Thirty-four percent of patients with probable cases who had a single positive agglutination test result also reported *Brucella* species antibodies detected by ELISA IgM or IgG testing, which cannot be easily interpreted without additional clinical or laboratory test data. Reports have described cross-reactivity for ELISA IgM tests in suspected brucellosis cases, leading to false-positive results (*35*,*36*). The general recommendation is to interpret the combined results for ELISA IgM and IgG testing, rely only on the ELISA IgG result, or conduct other laboratory testing for patient diagnosis. Because of the broad accessibility of ELISA testing and the frequency with which ELISA testing was reported among brucellosis cases in this study, CSTE incorporated ELISA IgG testing as a criterion for suspected case classification in the updated 2025 brucellosis case definition.

## Limitations

The findings in this report are subject to at least eight limitations. First, the identification, investigation, and reporting of brucellosis in the United States are conducted at the local level and can vary, which might affect the sensitivity and specificity of brucellosis surveillance. Cumulative incidence estimates were calculated using total cases over the study period and a single population denominator and therefore should not be interpreted as annual incidence. Second, the nonspecific symptoms of brucellosis can lead to misdiagnosis and underreporting. Third, although CSTE provides a standardized case definition, jurisdictions might adopt different definitions on the basis of local policies, which might lead to underreporting or overreporting. Fourth, in this analysis, only 49% of cases reported through NNDSS had accompanying brucellosis-specific supplemental information submitted to CDC, which might limit the assessment of *Brucella *species exposures. Improving the submission of the brucellosis supplemental CRFs is essential for enhancing surveillance and refining recommendations for brucellosis in the United States. Fifth, exposure and laboratory information often were missing from the CRFs received, limiting the data available for analysis. Reported exposures were descriptive and not mutually exclusive; in the absence of a comparison group, these data cannot be used to infer causality or quantify attributable risk. In addition, the substantial amount of data missing for hospitalization, death, and treatment variables limits interpretation of severity and treatment patterns. Sixth, brucellosis supplemental data submission to CDC includes manual methods such as fax and email, which are time-consuming. CDC is working to modernize data transmission processes to reduce the workload for jurisdictional partners and improve data availability (*37*). Supplemental case data are crucial for identifying exposures; without these data, the findings of this study are constrained. Seventh, missing information on negative laboratory tests, specimen types, and newer test methods (e.g., ELISA) limits the ability to evaluate overall *Brucella* species testing at a national level. Finally, the restrictions and costs associated with shipping select agents limit the amount and types of specimens received for *Brucella* bacteria testing at CDC. 

## Future Directions

Several changes implemented recently will likely affect CDC’s future brucellosis surveillance. First, the expansion of 1CDP and data modernization work at CDC now allows jurisdictions to submit supplemental CRFs directly through the platform rather than via fax, mail, or email. Second, as of January 16, 2025, *B. abortus*, *B. melitensis*, and *B. suis* are no longer regulated as select agents by the Federal Select Agent Program (*38*). This change will allow for more specimens to be shared with CDC, improving laboratory surveillance and information-sharing efforts. Finally, changes to the brucellosis case definition were approved by CSTE members in 2024, which will improve brucellosis surveillance at the jurisdictional and national levels (*31*).

## Conclusion

Improving brucellosis education among populations at risk for the disease could reduce exposure to *Brucella* bacteria and prevent brucellosis. Increasing awareness among providers and health officials, enhancing surveillance at the jurisdictional level, and improving how data are shared with CDC will help increase case ascertainment in the United States, which will guide future epidemiologic investigations and public health responses.

## Footnotes

* *Brucella* species can be classified by their lipopolysaccharide phenotype. Smooth *Brucella* species contain the O-polysaccharide (O-PS) and rough species do not. The presence of O-PS affects virulence with smooth species being more virulent (https://doi.org/10.1016/j.jiph.2023.01.002).

† 45 C.F.R. part 46.102(l)(2), 21 C.F.R. part 56; 42 U.S.C. Sect. 241(d); 5 U.S.C. Sect. 552a; 44 U.S.C. Sect. 3501 et seq.
